# Dysconnection Topography in Schizophrenia Revealed with State-Space Analysis of EEG

**DOI:** 10.1371/journal.pone.0001059

**Published:** 2007-10-24

**Authors:** Mahdi Jalili, Suzie Lavoie, Patricia Deppen, Reto Meuli, Kim Q. Do, Michel Cuénod, Martin Hasler, Oscar De Feo, Maria G. Knyazeva

**Affiliations:** 1 École Polytechnique Fédérale de Lausanne (EPFL), IC – School of Computer and Communication Sciences, Laboratory of Nonlinear Systems (ICLANOS), Lausanne, Switzerland; 2 Center for Psychiatric Neuroscience, Department of Psychiatry, Centre Hospitalier Universitaire Vaudois and University of Lausanne, Lausanne, Switzerland; 3 Department of Radiology, Centre Hospitalier Universitaire Vaudois and University of Lausanne, Lausanne, Switzerland; 4 Microelectronic Engineering, University College Cork, Cork City, Ireland; 5 Department of Neurology, Centre Hospitalier Universitaire Vaudois and University of Lausanne, Lausanne, Switzerland; University of Massachusetts Medical School, United States of America

## Abstract

**Background:**

The dysconnection hypothesis has been proposed to account for pathophysiological mechanisms underlying schizophrenia. Widespread structural changes suggesting abnormal connectivity in schizophrenia have been imaged. A functional counterpart of the structural maps would be the EEG synchronization maps. However, due to the limits of currently used bivariate methods, functional correlates of dysconnection are limited to the isolated measurements of synchronization between preselected pairs of EEG signals.

**Methods/Results:**

To reveal a whole-head synchronization topography in schizophrenia, we applied a new method of multivariate synchronization analysis called S-estimator to the resting dense-array (128 channels) EEG obtained from 14 patients and 14 controls. This method determines synchronization from the embedding dimension in a state-space domain based on the theoretical consequence of the cooperative behavior of simultaneous time series—the shrinking of the state-space embedding dimension. The S-estimator imaging revealed a specific synchronization landscape in schizophrenia patients. Its main features included bilaterally increased synchronization over temporal brain regions and decreased synchronization over the postcentral/parietal region neighboring the midline. The synchronization topography was stable over the course of several months and correlated with the severity of schizophrenia symptoms. In particular, direct correlations linked positive, negative, and general psychopathological symptoms to the hyper-synchronized temporal clusters over both hemispheres. Along with these correlations, general psychopathological symptoms inversely correlated within the hypo-synchronized postcentral midline region. While being similar to the structural maps of cortical changes in schizophrenia, the S-maps go beyond the topography limits, demonstrating a novel aspect of the abnormalities of functional cooperation: namely, regionally reduced or enhanced connectivity.

**Conclusion/Significance:**

The new method of multivariate synchronization significantly boosts the potential of EEG as an imaging technique compatible with other imaging modalities. Its application to schizophrenia research shows that schizophrenia can be explained within the concept of neural dysconnection across and within large-scale brain networks.

## Introduction

The hypothesis that schizophrenia (SZ) is a condition characterized by abnormal brain integration can be traced back to Bleuler, who emphasized that a splitting of the psychic functions (‘loosening of associations’) is a core problem in SZ [1]. The testable biological counterparts of such a clinical phenomenology of the disorder are anomalous structural integrity and/or functional connectivity of the brain.

The morphometric evidence in favor of the dysconnectivity model of SZ includes subtle but wide-spread morphological abnormalities observed in postmortem studies. Among supporting although indirect findings there are enlarged ventricles (reviewed in [2]), decreased cortical volume or thickness coupled with increased cell packing density [3], [4], [5], and reduced clustering of neurons [6]. The myelin of long-range connecting fibers can also be damaged in SZ [7]; also reviewed in [8], [9].

The *in vivo* neuroimaging studies largely confirm the reduced volume of cortical gray matter in SZ. In particular, associative areas including prefrontal, temporal, parietal, and limbic cortices are consistently found to be affected [10], [11], [12], [13]; for review see [14], [15], [16]. In line with this evidence, longitudinal studies revealed progressive loss of cortical gray matter in early-onset SZ [17], [18], [19], [20] .

A possible interpretation of these structural abnormalities is considered in the neuropil hypothesis [21], which claims that the reductions are caused by the pathological changes in the neuronal architecture and local circuitry. Yet the structural abnormalities seem to be quite subtle and were not replicated in a number of studies. That gave rise to another dysconnection hypothesis which emphasizes aberrant control of synaptic plasticity in SZ [22], [23]. However, the two hypotheses are not mutually exclusive, and both mechanisms should lead to cortical circuitry problems in SZ.

A necessary link between abnormal circuitry and basic SZ symptoms is functional connectivity. Following current views, by “functional connectivity” we understand cooperation between distributed neural assemblies in the brain. A common way of assessing the cooperation among cortical networks is measuring their synchronization by means of some deterministic (e.g. phase synchronization) or statistical (e.g. correlation) measure. Here, in agreement with deterministic dynamical systems theory, synchronization refers to the process by means of which two or more interacting subsystems adjust some of their temporal properties, i.e., synchronize their activities [24].

Synchronization as a measure of functional connectivity has been extensively used in the EEG studies of SZ. These studies, largely applying *bivariate* methods, e.g., (phase) coherence analysis of time series in pairs of EEG signals, exemplified abnormalities in EEG synchronization at rest and during the performance of cognitive tasks [25], [26], [27], [28], [29], [30], [31]. However, the limitations of bivariate synchronization analysis inevitably led to the region-of-interest approach that is an analysis of several pre-selected pairs of signals. In particular, based on a priori evidence, the synchronization abnormalities in SZ were largely tested for the EEG electrode pairs located over frontal, temporal, and parietal cortices, whereas reconstruction of the whole-head topography of synchronization remained unattainable.

Modern multichannel EEG techniques, combined with the advances in dynamical systems theory and in signal processing, allow a construction of *multivariate* synchronization measures readily translatable into synchronization maps. Indeed, recent work in nonlinear dynamical systems resulted in new application-independent multivariate measures of synchronization [32], [33], [34], [35]. Here, we address synchronization phenomena by means of the S-estimator, which, initially proposed for an EEG application [33], was also successfully applied to assess synchronization phenomena within other contexts such as cardio-encephalic-pulmonary interactions in anesthesia [36] and athletics electrocardiography [37].

The S-estimator exploits a theoretical consequence of cooperative (synchronization-like) phenomena in order to estimate the amount of synchronization within a set of measurements of arbitral cardinality [33], i.e., the fact that the portion of the visited state-space of two (or more) interacting dynamical systems is smaller than that visited by the same putatively non-interacting systems [38]. The S-estimator properties, including robustness with respect to measurement and dynamical noise, resiliency and scalability with respect to the number of measurements (channels), and sensitivity with respect to the amount of data (the length of measurements), were extensively tested [33], [39]. The S-estimator was proved to be a robust and easily scalable multivariate measure of synchronization, highly sensitive even with a reasonably small amount of data. Hence, it represents a perfectly suitable measure of the whole-head synchronization topography.

We applied the S-estimator technique to the resting state EEG with the aim of examining the whole-head landscapes of intra- and inter-areal functional connectivity in SZ patients. In this report we characterize the topography of the synchronization abnormalities in SZ. Furthermore, we show the relevance of this synchronization landscape to the clinical picture of SZ. Finally, we discuss our findings within the concept of neurodevelopmental dysconnection in SZ.

## Methods

### Subjects

Fourteen patients with mean age of 33.5±10.1 (here and henceforth mean values are presented with standard deviations) with schizophrenia or schizoaffective disorder were recruited from the in/outpatient schizophrenia units of the Psychiatry Department, Lausanne University Hospital. The group included 11 men and 1 lefthander. All diagnoses were made according to DSM-IV criteria on the basis of the Diagnostic Interview for Genetic Studies (DIGS) [40], or by a consensus of two experienced psychiatrists after a systematic review of medical records. Patients with a history of neurological illness or head trauma, with mental retardation (IQ below 60), or with a diagnosis of drug/alcohol dependence or abuse were excluded. Thirteen of them were receiving antipsychotic medication (12 atypical, 1 typical) at doses considered therapeutic by psychiatrists. Additional evaluations of psychopathology in patients included the Positive and Negative Syndrome Scale (PANSS) [41], which assessed the presence of symptoms within the same week as the first and the second EEG measurements.

Fourteen healthy control subjects (mean age 33.9±9.9) without known neurological or psychiatric illness or trauma and without substance abuse or dependence matched the patients for age, gender, and handedness. Eight controls were included based on the DIGS interview, and six controls based on the Symptom Checklist [42].

All participants were fully informed about the study and gave written consent. All the procedures conformed to the Declaration of Helsinki (1964) by the World Medical Association concerning human experimentation and were approved by the local ethics committee of Lausanne University.

### EEG recording and pre-processing

The EEG data were collected in a semi-dark room with a low level of environmental noise while each subject was sitting in a comfortable chair. The subjects were instructed to stay relaxed and motionless with eyes closed for 3–4 minutes. The resting state EEGs were recorded with the 128-channel Geodesic Sensor Net (EGI, USA). All the electrode impedances were kept under 30 kΩ; the recommended limit for the high-input-impedance EGI amplifiers is 50 kΩ. To keep the quality of recording under steady watch and to control vigilance in the subjects, the on-going EEG tracings were constantly monitored.

The recordings were made with vertex reference using a low-pass filter set to 100 Hz. The signals were digitized at a rate of 1000 samples/s with a 12-bit analog-to-digital converter. They were further filtered (FIR, band-pass of 1–70 Hz, notch at 50 Hz), re-referenced against the common average reference (CAR), and segmented into non-overlapping epochs using the NS3 software (EGI, USA).

Artifacts in all channels were edited off-line: first, automatically, based on an absolute voltage threshold (100 µV) and on a transition threshold (50 µV), and then by thorough visual inspection, which allowed us to identify and reject epochs or channels with moderate muscle artifacts not reaching threshold values. The optimal artifact processing strategy depends on the nature of the EEG features under analysis. Since interpolation adds a common component to signals at different electrodes, it may artificially increase synchronization measures. Therefore, we took a conservative approach by excluding from further analysis the sensors that recorded artifactual EEG in at least one subject. Finally, 100 sensors were used for further computation. Data were inspected in 1 s epochs and the number of artifact-free epochs entered into the analysis was 185±51 (first EEG) and 164±35 (second EEG) for the patients, and 195±45 for the control subjects.

Estimates of synchronization depend on the EEG reference [43]. For the dense array EEG, the CAR was shown to be an optimal choice [44]. Furthermore, in our recent studies we demonstrated that interhemispheric coherence computed for CAR EEG signals reliably correlates with the fMRI activation of neural assemblies presumably involved in synchronized activity [44], [45].

### Measure of Synchronization: S-estimator

The S-estimator exploits a theoretical consequence of synchronization phenomena to indirectly assess and quantify the synchronization (cooperativeness) within a set of measurements of arbitral cardinality [33]. In a network of interacting dynamical systems, the observable dimensionality (*embedding dimension*) of the whole dynamical network decreases in consequence of the interactions amongst its elements [38].

For example, let us consider a very simple dynamical network of two planar pendula. According to Newtonian mechanics, each of them has dynamics of dimension two, given by their respective positions and velocities. By considering them together, the whole network has putative dimension four. However, as already noticed by Huygens back in 1665, if we couple them, they may eventually oscillate together (perfectly synchronized). In this case, the “observable” dimensionality of the whole network is only two. In fact, of all the possible four-dimensional state combinations (positions and velocities of the two pendula), the trajectories of the two synchronized pendula visit only the subpart where the two speeds and two positions are equal to each other, which is a two-dimensional subset of the whole four-dimensional state-space.

The S-estimator indirectly measures the synchronization-induced contraction of the embedding dimension by determining the dispersion (entropy) of the eigenvalues of the correlation matrix of a multivariate set of measurements. In formulae, let us consider a *P*-variate time series 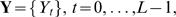
where *Y_t_*∈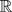 *^P^* is the *t*-th sample observation vector and *L* is the number of available samples.

Let us also assume that **Y** has been mean-detrended and normalized to unitary variance, which, without any loss of generality, makes the synchronization measurement independent of the relative amplitudes of the signals. For the given time series **Y**, the S-estimator is defined as
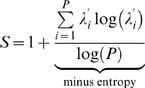
(1)where *λ′_i_* = *λ_i_*/*P* designates the normalized eigenvalues of the correlation matrix of the multivariate time series **Y**.

This definition applies when considering the measured time series without any embedding (reconstruction of the state-space). However, if we want to account for the state-space trajectory through a suitable embedding, we need to proceed in two steps: first, we reconstruct, from the measured time series, the trajectory of the dynamical phenomena under observation by means of delay embedding [46]; second, we compute the S-estimator, as defined by Eq. (1), in the reconstructed (extended) state-space. However, in this case a preliminary normalizing step of the correlation matrix is necessary [47]. As a consequence of Eq. (1), the S-estimator quantifies the amount of synchronization within a data set by implicitly comparing the actual dimensionality of the set with the expected full dimensionality of the asynchronous set.

To understand how the entropy (dispersion) of the eigenvalues of the correlation matrix relates to the dimensionality of the dynamical phenomenon behind the observation, it is sufficient to resort to linear algebra [48]. In fact, according to the Singular Value Decomposition, the eigen-decomposition of the correlation matrix provides a linearly transformed coordinate system for the original time series **Y**. In Principal Component Analysis (PCA) this new coordinate system is used to compute the so-called population of principal components. Indeed, when performing PCA, a given multivariate time series is transformed into the principal components by a linear transformation that projects the original time series into the eigen-base of the correlation matrix of the time series itself. In this new coordinate system, each normalized eigenvalue gives the relative importance of its corresponding eigen-direction, namely how much this eigen-direction (which is one of the system's dimensions) is visited by the observed trajectory [49].

Consequently, the entropy of the normalized eigenvalues of the correlation matrix accounts for how many dimensions are significantly visited by the observed trajectory. Indeed, when all the normalized eigenvalues are roughly of the same value (maximal dispersion of eigenvalues), all the state-space dimensions are almost equally visited. In this case the entropy of the eigenvalues is maximal (close to 1), therefore S is close to 0, meaning no contraction of the embedding dimension (that is, no synchronization). Alternatively, when all the normalized eigenvalues are roughly 0 and only a few of them are appreciably nonzero (minimal dispersion), only a few state-space dimensions are visited. In this case the entropy of the eigenvalues is minimal (close to 0), consequently S is close to 1, meaning maximal contraction of the embedding dimension, and thus strong synchronization.

### Assessment of the whole-head topography of synchronization

The changes in the whole-head S-maps associated with SZ were assessed through a systematic analysis procedure consisting of three main steps, which are described in detail below.

#### Normalization

In order to make the synchronization measure independent of the relative amplitudes of the signals, the pre-processed (filtered, segmented, and CAR referenced) EEGs were, first, mean-detrended and normalized to unitary variance.

#### Computation of the synchronization topography

**Figure 1:**
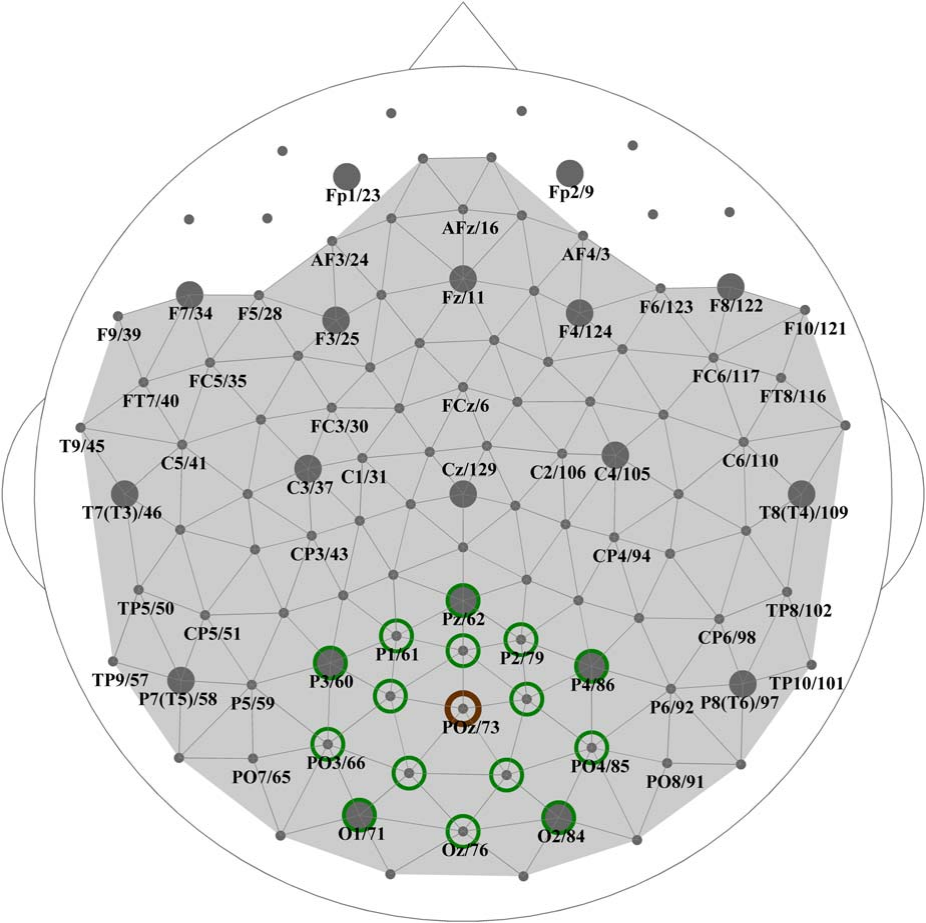
Head diagram of the EEG sensor positions and labeling. The diagram shows the correspondence between the high-density 129-channel Sensor Net (EGI, Inc.) and the International 10–10 System. The Sensor Net locations that match the positions of International 10–10 system are labeled. The 10–10 System names are followed by the numbers of the Sensor Net. The sensors corresponding to the 10–20 System (presented in all the maps hereafter) are in bold. The gray background highlights all the sensors included in the analyses. The sensor locations encircled in green exemplify the first and second neighborhoods for the sensor encircled in brown (sensor 73), i.e., the territory considered in the calculation of a single value of S-estimator.

For each sensor, the S-estimator has been computed epoch-wise over the cluster of locations defined by the sensor itself and the surrounding sensors that belong to its first- and second-order neighborhoods [33]. Such a cluster (on average about 12 cm wide) is shown in Fig. 1 for occipital sensor 73. The whole-head maps for individual subjects were acquired by computing the S-estimator iteratively on a sensor-by-sensor basis. These instantaneous (epoch-wise) S-maps were collapsed by averaging across all artifact-free epochs, obtaining in this way a subject's typical whole-head topography of synchronization.

To detach the assessment of the S-landscape from the general (individual) level of synchronization, each map was relativized to its average value, i.e., the latter was subtracted from each local S value. Finally, the *subjects' relative maps of synchronization* were collected into two populations (patients and controls), of 14 members each, to be considered in the next step of analysis.

#### Statistical analysis

As follows from its mathematical definition, the values of the S-estimator are bounded within the interval [0,1], and thus they cannot be normally distributed. Since the non-normal distributions are better described by their medians than by their means, the medians for the two samples entered into statistical analysis.

More precisely, the topographies of the two populations were compared sensor-wise by means of the *signtest* for paired samples [50]. For each sensor, the signtest assessed whether the medians of the two matched populations of controls and patients (assumed to have arbitrary and continuous distributions) were different or not. In this way we obtained the centers of clusters for which the S-estimator of the patients' population was significantly higher or lower than that of the controls' population. The interhemispheric asymmetry in both groups was assessed similarly for each pair of symmetric sensors.

Since the topographies of the two populations were compared sensor-wise (independently for each sensor), in order for the maps to have statistical sense as a whole, the P-values of each comparison needed to be corrected for multiple comparisons. As the computation of each S-value involved several sensors from the neighborhood (see above), the P-values of the sensor-wise comparisons were corrected by means of the BH false discovery rate method [51], taking into account the uncorrected P-values of each sensor's first- and second-order neighbors. The BH-corrected significant P-values were verified to have at least P<0.05.

All the computations mentioned here and afterwards were performed within the Matlab environment: the synchronization was estimated using the S-estimator toolbox, while the statistical analysis was performed by means of Mathwork's official statistic toolbox. The S-estimator toolbox is available gratis at http://aperest.epfl.ch/docs/software.htm. Further information is available at http://www.mathworks.com/access/helpdesk/help/toolbox/stats/.

### Assessment of temporal stability of the S-maps

For 10 of the 14 patients the EEG data were obtained during two recording sessions with a 2–4 month interval between them. The S-maps for the second EEG were computed according to the procedure described above. To test the temporal stability (i.e., repeatability) of S-maps, we performed the following three-step analysis.

First, the S-maps based on the second EEG were compared to the controls' data, namely to the subgroup of 10 matching controls. To this end, we performed the signtest-based procedure as described above. For fair assessment of the repeatability of the synchronization pattern, we also recomputed the difference S-map based on the first EEG for the same 10 patients.

Second, again using the signtest-based procedure, we tested whether any particular pattern emerges when comparing the patients' synchronization topography based on the first vs. second EEG.

Third, we assessed the hypothesis that the topographies based on the first and the second EEG are spanned by the same distribution. For that we computed sensor-wise the P-values of the *two-sample Kolmogorov-Smirnov goodness-of-fit hypothesis test* [50] and corrected them according to the BH false discovery rate method. Note that, although the asymptotic P-values of the Kolmogorov-Smirnov test become very accurate for large sample sizes, they are still reasonably accurate for sample sizes N_1_ and N_2_ such that (N_1_N_2_)/(N_1_+N_2_)≥4 [50], which is indeed the case for our sample, where N_1_ = N_2_ = 10, and therefore the ratio is 5.

### Correlation analysis

In order to assess to which extent the abnormalities in synchronization topography are related to the clinical picture of SZ, we correlated the changes in S-estimator in patients to their scores on the Positive Symptom Scale (PS), the Negative Symptom Scale (NS), and the General Psychopathology Scale (GP).

More precisely, for each patient we determined the relative quantitative changes of synchronization (ΔS) by subtracting sensor-wise the respective control group average from the patient's average. These ΔS values were correlated with the clinical scores by means of the Pearson Product Moment Correlation. The BH correction for multiple testing was applied to P-values of correlation coefficients.

For assessing the interhemispheric asymmetry of these correlations, the leave-one-out algorithm was used [52]. That is to say, the correlation topography was determined 14 times, each time dropping one patient and making the calculations for the remaining 13 patients. The asymmetry for the resulting 14 correlation topographies was assessed with the signtest and BH corrected.

### Comparative analysis of the EEG power and synchronization topography

Though the viable precautions to reduce the effects of volume conduction were taken (see EEG recording and pre-processing and Assessment of the whole-head topography of synchronization), the theoretical possibility that the differences between patients' and controls' synchronization topographies could be a side effect of differences in signal-to-noise ratio, rather than being related to effective synchronization, still remains.

To figure out whether such a possibility could be the case, we compared the topography of quantitative changes in synchronization with the topography of quantitative changes in EEG power.

To this end, we computed power maps for individual patients and controls for the broad-band EEG (1–70 Hz). More precisely, the topography of power was estimated via i) computing, sensor-wise and epoch-wise, the power spectral density by means of Welch's averaged modified periodogram [53]; ii) averaging the spectra across epochs; and iii) integrating the spectra over the whole frequency range. The resulting individual average power topographies were used to compute absolute and relative quantitative changes of power in patients. Finally, for each sensor of each patient, we computed the Pearson correlation coefficients between the relative changes in EEG power and synchronization. This procedure was completely analogous to the one used for assessing the relationships between the topographical changes of synchronization and the SZ symptoms. P-values were corrected by means of the BH false discovery rate method.

## Results

### Mapping the synchronization landscape in SZ patients and in controls

**Figure 2:**
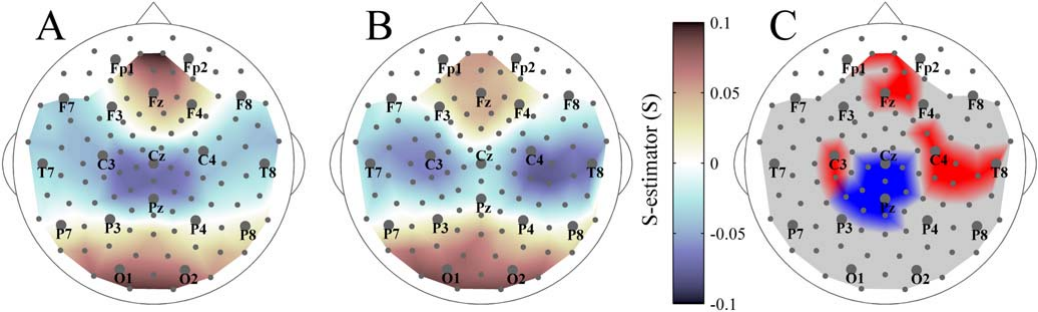
Whole-head S-estimator maps for SZ patients and normal controls. Group-averaged maps for the broad-band resting EEG (1–70 Hz) are shown for patients (A) and controls (B). C: Difference map, Patients vs. Controls. Here and hereafter the clusters of sensors with S-estimator significantly higher or lower in patients than in controls are in red or blue, respectively. There are no significant differences in the gray regions.

**Figure 3 pone-0001059-g003:**
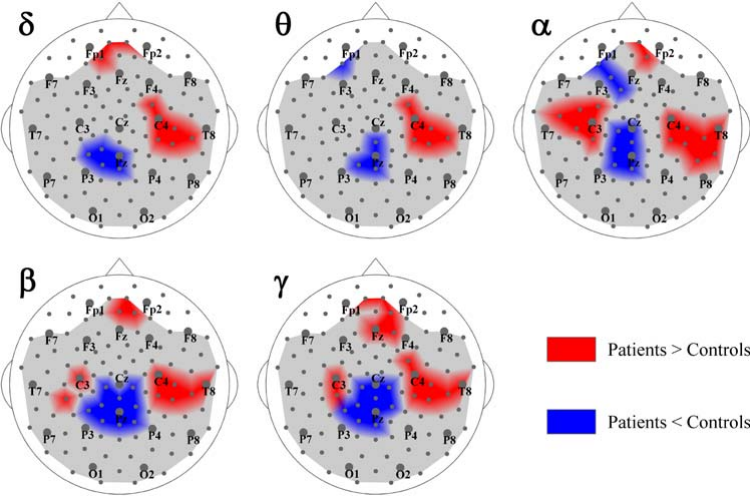
Spectral breakdown of the S-estimator data into conventional EEG bands. Group-averaged difference S-maps (Patients vs. Controls) are given for the following EEG bands: δ: 1–3 Hz; θ: 3–7 Hz; α: 7–13 Hz; β: 13–30 Hz; and γ: 30–70 Hz.

Figures 2 and 3 show group-averaged whole-head S-maps for the broad-band EEG (1–70 Hz) and for conventional EEG frequency bands including delta (1–3 Hz), theta (3–7 Hz), alpha (7–13 Hz), beta (13–30 Hz), and gamma (30–70 Hz). In these maps, the S-value (or a significant variation in S-values between patients and controls in the difference maps) assigned to a single sensor as a color spot over this sensor represents the synchronization of a larger region, an example of which is presented in Fig. 1.

With this in mind, let us consider the S-maps for the broad-band EEG obtained for the groups of 14 patients and 14 controls (Fig. 2A, B). They reveal a distinct pattern of regional synchronization in the schizophrenia patients. Indeed, the difference map for the broad-band EEG (Fig. 2C), where only significant differences (P<0.05, with BH correction) are depicted, confirms this observation.

The synchronization landscape in SZ is characterized by hyper-synchronization significant for 3 centro-parietal sensors (corresponding to the C3, CP3, and CP5 locations of the extended 10–20 system) over the left hemisphere and for a large cluster of 10 sensors over the right hemisphere. This latter cluster is limited by FC locations anteriorly, by C2 and CP2 medially, and by P4–P8 posteriorly, and extends until the last row of sensors (T8, TP, and TP10) laterally. Therefore, at rest, synchronization across fronto-centro-temporal locations in the left hemisphere, and over fronto-centro-temporo-parietal locations in the right hemisphere, is higher in SZ patients than in controls.

At the same time, we found a midline cluster (13 sensors) of hypo-synchronized locations over the centro-parieto-occipital region that also distinguishes the patients from control subjects. This cluster was located roughly between the coronal C and OP rows and limited laterally by the first parasagittal rows of sensors (according to the extended 10–20 system). Considering that the second neighborhood covers a territory with a radius of about 6 cm, this cluster represents reduced synchronization both between and within hemispheres.

The S-landscapes for separate EEG frequency bands are shown in Fig. 3 (all differences are significant at least at P<0.05 with BH correction). In general, the narrow-band variations between patients and controls follow the pattern revealed for the broad-band EEG. Yet the differences are more pronounced for the higher frequency bands (alpha-gamma range). This is especially true for the left hemisphere as there is no significant S-increase in the delta- and theta-bands. The posterior midline region, characterized by S-decrease, is also reduced at low frequencies. Similarly, the frontal right hemisphere cluster close to the midline shows an S-increase only across higher frequency bands. Hence, collectively, these facts point to the broad-band nature (at least within the range of higher frequencies) of the variation in the synchronization landscape in SZ.

The S-maps in Fig. 2 and 3 present apparently asymmetric patterns of significant differences between SZ patients and controls, which could result from an asymmetric topography of synchronization in either group. We tested both possibilities by comparing S-values from symmetric sensors, but failed to confirm significant interhemispheric asymmetries for either group (P>0.05 BH corrected).

### Whole-head S-maps in SZ patients: a replication

**Figure 4:**
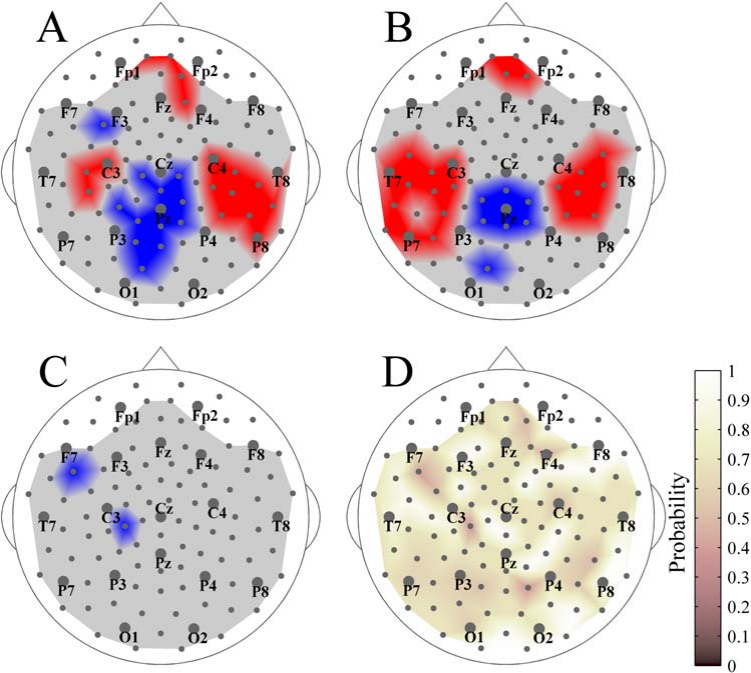
Temporal stability of the S-estimator topography in SZ. Group-averaged difference maps (Patients vs. Controls) for the broad-band EEG from ten patients who participated in the first (A) and second (B) EEG sessions. C: Difference map between the first EEG vs. second EEG of patients. The regions where the S-estimator in the patients' first EEG was significantly higher or lower than that in the second EEG are in red or in blue, respectively. There are no significant differences in the gray regions. D: Map of the likelihood that the synchronization estimations from the first and second EEG can be considered stationary according to *Kolmogorov-Smirnov* test. The color is inversely related to the probability: the lighter, the more probable.

In the SZ literature, the resting state EEG is predominantly assumed to be a stable individual parameter, the variations of which reflect certain pathological traits in SZ. However, the absence of consistency in the results (see Discussion) might be attributed to the impact of the situation-dependent EEG features. To test the reliability of the S-estimator as a measure of SZ-associated traits, we repeated the EEG recordings in 10 patients with 2–4 month intervals. Their group-averaged difference maps (Patients vs. Controls) computed for the first and the second EEGs were qualitatively similar (Fig. 4A, B).

The maps shown in Fig. 4C, D confirm that the deviation in the SZ synchronization topography is a stable feature. Indeed, for the vast majority of locations no significant differences (patterns) emerged when comparing the patients' S-maps derived from the first and second EEG sessions (Fig. 4C), although S-estimator values for two sensors (35 and 38 located near C3 and F7, respectively) varied significantly (P≈0.05 BH-corrected). Furthermore, a sensor-by-sensor correlation analysis between the matched S-values from the first and second EEG session reported very high correlation values (Pearson's *r* = 0.789±0.142, P<0.01 BH-corrected). Finally, according to the *Kolmogorov-Smirnov test* (Fig. 4D), patients' S-values from the two EEG sessions are highly likely (P = 0.791±0.129 BH corrected) to belong to the same distribution. Interestingly, all the maps in Fig. 4 reveal a tendency of the S-estimator to have higher temporal stability over the right hemisphere and over the posterior regions.

### Correlation between S-estimator and SZ symptoms

As the replication experiment showed, the stable features of the synchronization pattern in SZ included the hyper-synchronization across the temporal lobes and adjacent cortical territories and the hypo-synchronization of the EEG from posterior sensors close to the midline. Assuming that these changes are SZ-associated, we hypothesized a correlation between the severity of clinical SZ-symptoms and the magnitude and direction of S-changes. Therefore, for hyper-synchronized temporal clusters we expected direct correlations: the higher the synchronization increase, the greater the symptoms. The same logic suggested inverse correlations for a midline cluster of hypo-synchronized sensors.

**Figure 5:**
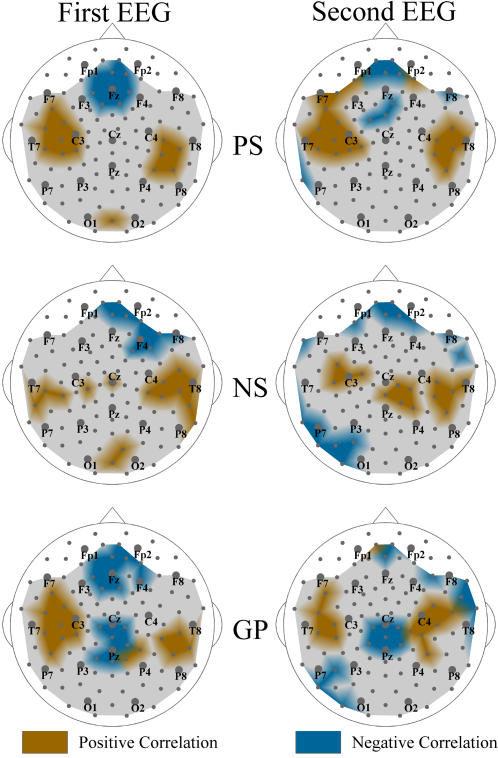
Correlation between synchronization and SZ symptoms. The topographies of correlations between the S-estimator changes in patients and their symptoms as measured by the Positive Symptom Scale (PS), Negative Symptom Scale (NS), and General Psychopathology Scale (GP) are shown. The regions where the significant correlations are direct or inverse are marked in brown or turquoise, respectively. There are no significant correlations in the gray regions.

The correlation analysis was performed sensor-by-sensor and included all the sensors used for the S-estimator computation (see Methods for details). The correlation maps showing sensors for which Pearson's correlations (*r*) reached a significance level of P<0.05 (BH-corrected) are presented in Fig. 5. As can be seen, the sensors having significant direct correlation between S-changes and total PS scores form two asymmetric clusters that overlap the hyper-synchronized clusters over the temporal regions shown in Figs. 2–4. Notably, the left correlation cluster includes all the sensors with significantly hyper-synchronized EEG, but spreads anteriorly much farther, to the sensors of the coronal F-row. Its topography reproduced itself well after a 2–4 month period (cf. the “First EEG” and “Second EEG” columns in Fig. 5). The mean *r* values for this cluster were 0.62±0.07 for the first EEG and 0.57±0.05 for the second one. The cluster of significant correlations in the right hemisphere also overlapped the location of hyper-synchronized temporal sensors. Similar to the left hemisphere, mean *r* values for this cluster were equal to 0.57±0.07 for the first EEG and to 0.61±0.07 for the second one.

The correlation maps for the total NS scores also revealed bilateral clusters, but repeatable topography was evident only over the right hemisphere. This cluster overlapped the hyper-synchronized group of sensors to a great degree for both EEG sessions. The mean *r* values were 0.68±0.08 and 0.59±0.06 for the first and second EEG, respectively. Over the left hemisphere, the correlation clusters neither considerably overlapped with the hyper-synchronized ones nor replicated themselves in the second EEG.

GP scores showed a somewhat different topography of correlations. Along with bilateral direct correlations (0.56±0.05 and 0.58±0.08 over the left, and 0.58±0.04 and 0.61±0.06 over the right hemisphere for the first and the second EEG, respectively) located roughly over the same region as shown for PS, we found significant inverse correlations in the hypo-synchronized midline region. They point to the fact that the severity of psychopathological problems increases with the reduction of the midline synchronization. We replicated this cluster in the second EEG and the mean *r* values for the cluster were, respectively, −0.61±0.09 and −0.59±0.07.

Unexpectedly, for each syndrome scale, we also found and replicated a cluster of frontal locations with inverse correlations, showing that the milder symptoms correspond to greater hyper-synchronization. In particular, the PS and GP symptoms correlated in locations close to midline. The *r* values varied between −0.55±0.05 and −0.53±0.04 for the first and second EEG, respectively. The frontal cluster of inverse correlations with NS mostly belonged to the right hemisphere (−0.54±0.02 and −0.60±0.06, first and second EEG, respectively).

The interhemispheric asymmetry of correlation topography that can be seen in Fig. 5 exists as a trend in our data, since we could not confirm it with rigorous statistical testing (P>0.05 BH corrected).

### The relationship between S-estimator and EEG power

As other measures of synchronization [54], [55], the S-estimator is affected by the amplitude of the EEG signals. Both differences in the power of a signal and differences in synchronization among sources distributed under a sensor and its neighbors can result in S-estimator changes. That is why we supplemented our synchronization study with the analysis of the EEG power differences between the SZ patients and controls.

**Figure 6:**
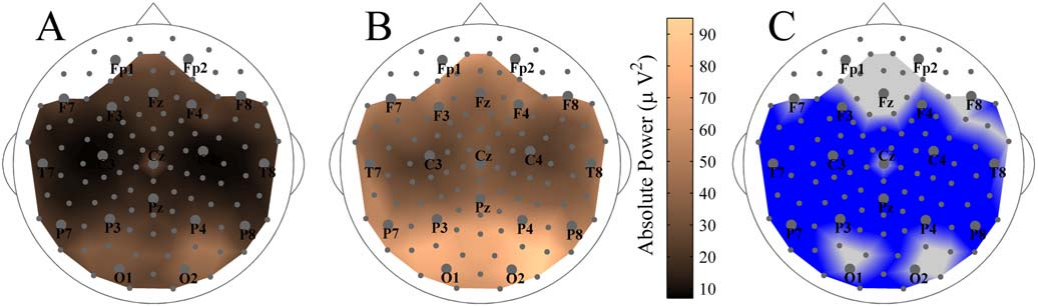
Whole-head power maps for SZ patients and normal controls. The group-averaged maps of absolute power for the broad-band resting EEG are shown for patients (A) and for controls (B). C: Difference map (Patients vs. Controls). In the blue regions the absolute power in patients is significantly lower than that in controls. There are no significant differences in the gray regions.

**Figure 7:**
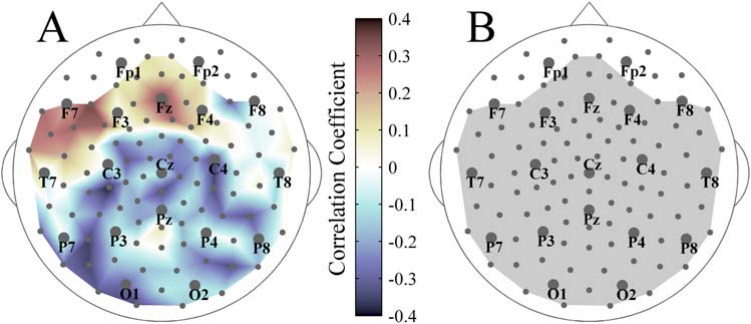
Relationship between synchronization and power. A: Correlation map between the relative changes of S-estimator and the relative changes of power for patients. The correspondence between color and correlation strength is shown by the scale bar. B: Significance map for the correlations shown in A; color convention is as in Fig. 5.

The whole-head maps of absolute power for the broad-band EEG from patients' and controls' populations are shown in Fig. 6A and B, respectively. As illustrated in the difference map (Fig. 6C), the power of EEG was generally lower in the patient group (P<0.02 uncorrected and P<0.05 BH-corrected). Only a few sensors, including the midline frontal region (Fz and its neighbors) and bilateral occipital clusters including O1 and O2 locations, did not show significant power reductions. These differences in power, uniform over the major part of the head surface, cannot account for the S-estimator topography. Indeed, clusters with both increased and decreased S were located within the large regions of reduced EEG power. Furthermore, as Fig. 7 shows, there is no significant correlation between S-changes and power-changes in patients. The *r* values are 0.18±0.11 for direct and −0.19±0.10 for inverse correlations as illustrated in Fig. 7A. Indeed, there is no single sensor with a significant correlation (Fig. 7B).

## Discussion

### Multivariate S-estimator maps and their relevance to the bivariate measurements of synchronization in SZ

Here we report the first application of a new method of EEG analysis to schizophrenia research–a method that determines EEG synchronization by relating it to the shrinking of the state-space embedding dimension. The whole-head mapping of multivariate synchronization with S-estimator revealed a specific synchronization landscape in schizophrenia patients. Its most prominent features include increased synchronization over temporal and frontal brain regions and decreased synchronization in the cluster of post-central locations neighboring the midline. Therefore, the S-maps do not support a simplistic view of schizophrenia as a hypoconnectivity disorder, but demonstrate a novel aspect of the abnormalities of functional connectivity: namely, their regional specificity. This pattern appears to be reproducible across conventional EEG frequency bands and to be relatively stable over time at least over the course of several months.

The results of multivariate and bivariate methods are not directly comparable, since they approach different aspects of the synchronization phenomenon. Nevertheless, being used in the analysis of the same data, various measures of synchronization detect coupling, although with different sensitivity [33], [56]. In particular, the S-estimator and spectral coherence analyses yield similar results (cf. [33], [44]). With this in mind, we turn to the qualitative comparison of our results with that from preceding studies.

The analysis of the resting-state EEG synchronization in SZ with bivariate methods has resulted in quite a mixed picture, which includes both increased and decreased synchrony between a few pre-selected pairs of EEG signals. Increased EEG coherence values in SZ patients have frequently been shown occurring both intra- [57], [58], [59] and inter-hemispherically [58], [60]. Yet a reduction in coherence has also commonly been reported [61], [62], [63], [64], [65]. Unfortunately, the coherence estimates obtained with the common vertex or linked ears references, as was the case for some of these reports, are difficult to interpret because of the problems outlined in Methods. Moreover, our synchronization estimates do not include long-distance connections (>12 cm). That leaves us with only several qualitatively compatible papers reporting (phase) coherence for CAR, bipolar, or Laplacian EEG.

In a broad sense, the hyper-synchronized temporal clusters shown here are consistent with the intrahemispheric coherence increase shown previously for the pairs composed by frontal, central, temporal, and parietal electrodes [57], [58], [59]. Furthermore, a common trend is revealed by the S-estimator decrease in the parietal midline cluster and by the reduction in inter-hemispheric coherence reported earlier [61], [62], [64], [66].

Yet it should be noted that the S-maps reported here present overwhelmingly more detailed evidence of the surface topography of synchronization than bivariate measurements do. Such maps of abnormal regional coordination among the neurophysiological processes distributed across cortical areas might greatly boost the potential of EEG as a diagnostic tool, provided that they correlate with the fundamental features of SZ.

### S-estimator maps and the clinical picture of SZ

We have chosen to correlate S-maps with the PANSS which is a conventional diagnostic tool, although there are disadvantages to this choice. In particular, the PANSS subscales represent constellations of various features, the brain counterparts of which might partially overlap. That does not allow us to reveal the full potential of the state-space mapping of EEG synchronization for topographically dissociating the brain sources underlying SZ. Yet this drawback is outweighed by the advantages of addressing the summarized picture of SZ and of obtaining results compatible with previous observations.

According to our findings, the severity of both positive and negative symptoms directly correlates with the S-increase within the hyper-synchronized temporal clusters and in their neighborhood (cf. Figs. 2–4, and 5). Although we found bilateral correlations for both scales, the positive symptoms show a more stable pattern over the left hemisphere, while the negative symptoms reveal a repeatable pattern over the right hemisphere.

These findings are consistent with different lines of evidence relating left-side temporal lobe abnormalities to the positive type of symptoms. Specifically, the reduction of the entire volume of the superior temporal gyrus correlates with the positive syndrome [67], [68], whereas the decrease of its anterior part correlates with auditory hallucinations [69]. The abnormalities of regional cerebral blood flow in the left temporal lobe might also be related to the positive symptoms in SZ [70]. Interestingly, the SZ-like psychoses with positive symptoms in epilepsy are associated with the left temporal lobe lesions [71]. Electrical stimulation of the superior temporal gyrus (STG) results in auditory hallucinations and disordered thinking [72].

Recent findings specifically support both the increase in temporal connectivity and its association with positive symptoms in SZ. In patients with hallucinations, a diffusion tensor imaging study [73] found increased white matter directionality in the arcuate fasciculus compared with controls or patients without hallucinations, these differences being most prominent in the left hemisphere. Thus, our findings are both consistent with and complementary to the evidence reported previously. They suggest that functional intra-hemispheric hyper-connectivity might be the basis of the positive symptoms.

The correlation pattern between the S-estimator and the negative symptoms is consistent with current knowledge of the relationship between the underlying functional deficits and the functional specialization of the right hemisphere. This refers to such functions as perception and/or expression of affect controlled by the right hemisphere [74], [75], [76] and compromised in SZ [77]. This is also valid for the higher-order language functions including discourse planning and comprehension, understanding humor and metaphors, and generation and comprehension of emotional prosody mediated by the right hemisphere [78]. They are essential for social communication, which is also impaired in SZ [78], [79], [80].

Although the negative symptoms are mostly associated with the frontal lobe changes (see further in this Discussion), recent imaging studies support the involvement of the bilateral or right-hemisphere temporal structures. Among them is an MRI study that found a bilateral reduction of STG gray matter in SZ patients with predominantly negative symptoms [81]. A positron emission tomography (PET) study showed that patients with mainly negative symptoms had lower metabolic rates in the right hemisphere, especially in the temporal and ventral prefrontal cortices, compared both to patients with positive symptoms and to normal subjects [82].

The General Psychopathology Scale showed a somewhat different topography of correlations. Along with bilateral temporal clusters of direct correlations similar to those found for the positive syndrome, we demonstrated inverse correlations within the hypo-synchronized postcentral midline region (cf. Figs. 2–4, and 5), which point to the fact that the severity of the symptoms increases with a decrease in synchronization. This is not surprising, considering that in SZ many fundamental psychotic features, including the lack of awareness, impaired control of actions, poor attention, increased reaction times, etc., are associated with the abnormal functioning of the superior parietal cortex [83], [84].

In contrast to the intrahemispheric temporal and midline postcentral clusters, the frontal correlations shown here were omnipresent across all the types of symptoms, and, surprisingly, revealed an inverse relationship between the severity of SZ symptoms and synchronization abnormality. Although such counter-intuitive links were previously found between clinical improvement and medial frontal gray matter loss [19], or between clinical improvement and hemisphere volume reduction [85], more observations are required for a meaningful interpretation of these data.

On the whole, with the correlation analysis, we confirmed the clinical relevance of the S-estimator maps. Specifically, the topography of correlations overlapped with the topography of synchronization changes in SZ patients compared to the control subjects. Furthermore, the surface topography of correlations appeared to be relevant to the brain regions known or suspected to be involved in the pathological process. However, the relationship between surface maps and underlying cortical pathology requires further consideration.

### Methodological aspects of the state-space analysis of EEG

To interpret the surface S-maps in the meaningful terms of neurophysiology, we must answer the two principal questions: i) What kind of phenomena does the S-estimator measure?, and ii) what is the relationship between the surface S-map and the underlying brain functional topography? Due to the limitations inherent in the EEG technique, neither question has a general answer: there are different scenarios that could result in similar changes in S-estimator, and there is no one-to-one relationship between surface and brain topography. At the same time, within the frame of our study, both questions can be provisionally answered based on a priori knowledge and supplementary analyses.

With respect to the first question, the EEG potentials measured over the scalp represent a combination of regional, local, and global sources [55], [86]. Due to the sensitivity profile, the surface EEG potentials are mostly generated by the radial sources of large dipole layers in the gyral crowns [86]. Depending on the EEG technique, either local (with Laplacian EEG) or regional-to-global potentials (CAR EEG recorded with a high-density array of sensors) can be analyzed.

The resting state EEG is generated largely by regional-to-global sources, which, in an activated brain, give way to predominantly local sources [87]. Furthermore, given subtle but wide-spread differences in the cortical tissue shown by neuroimaging methods (see the next paragraph), it is reasonably safe to expect that the SZ-associated changes emerge in the extended dipole layer that belongs to the surface cortical areas affected by the disease. With this in mind, we adopted the common-average reference EEG signals for computing the S-estimator. However, while providing measurements at an appropriate spatial scale, the CAR potentials are impacted by the volume conduction effects [86].

For that reason, we need to distinguish between possible sources of the S-estimator changes. In principle, both differences in the power of EEG signals and in the cooperative behavior of distributed neural networks can result in synchronization changes. S-estimator, as other measures of synchronization [54], [55], is affected by EEG power. However, our supplementary analysis showed that the topography of power differences did not match the topography of S-estimator differences, and, moreover, there were no correlations between the power and S-estimator between-group differences either within the clusters of S-changes associated with SZ or outside of them. These findings strongly suggest that there are true changes in synchronization and/or cooperativity behind the S-estimator differences.

The second question, regarding the brain topography behind S-maps, can be tentatively answered using a priori knowledge, including invaluable data from the neuroimaging methods with high spatial resolution. Indeed, given both EEG properties *per se* and anatomical findings in SZ, extended superficial gyral surfaces are the most likely source of the synchronization changes between SZ patients and controls.

### S-maps in SZ vs. maps from other neuroimaging modalities

The main elements of the S-landscape in SZ appeared to be the central-to-parietal midline hypo-synchronized region together with frontal and temporal hyper-synchronized regions. The S-maps appeared to be stable in time and similar across the EEG frequency bands, suggesting structural brain changes in SZ as a putative basis of synchronization changes. Since EEG is assumed to be generated due to the modulations of large-scale synaptic action fields, defined by the numbers of active excitatory and inhibitory synapses per unit volume of tissue [87], changes in potentials recorded from the head surface can be connected to the abnormalities of the brain tissue that affect synapses.

Abnormalities of gray matter in SZ have been repeatedly described in the literature. Typical findings consist in the reduction of gray matter and/or an increase of neuronal density pointing to the decline of neuropil (see Introduction). Some of the affected regions that have been most frequently reported in the SZ literature occupy the large convexital surface located under and close to the hyper-synchronized clusters shown here.

These regions include the superior frontal [88], [89], [90], inferior frontal [88], [91], and superior temporal gyri [69], [88], [92], [93]. Due to their surface location, these gyri should be a powerful source of EEG signals. Indeed, the landscape of S-estimator changes is strikingly similar to the maps of gray matter loss in patients with early-onset SZ that reveal involvement of the temporal, dorso-lateral prefrontal, and dorsal centro-parietal cortices [89].

In particular, the dorsal hypo-synchronized cluster probably captured changes of functional activity in the central-to-parietal cortex. The parietal cortex has been less intensively imaged and with inconsistent results (reviewed by [16]), although recent studies point to a subtle reduction of parietal volume including that of the superior parietal gyrus [13], [19] and the postcentral gyrus [89]. Among other midline abnormalities documented in SZ [94], [95], the changes of the corpus callosum (CC) could affect S-estimator, since CC defects result in an inter-hemispheric synchronization decrease detectable in the resting EEG [96]. As the medial cortical surface contains largely tangential sources, their impact on the surface EEG is unlikely to be crucial. Nevertheless, we cannot exclude sources in the precuneus and cingulate gyri, which showed gray matter reductions in SZ [13], [19].

Therefore, on a large scale, the synchronization topography obtained with the S-estimator method is consistent with the imaging results from other techniques. At the same time, the directional specificity of the S-estimator changes, including increased synchronization in the temporal and frontal clusters and decreased synchronization in the midline centro-parietal cluster, came as a surprise. Given the restrictions of the EEG approach, we can only conjecture as to why SZ-associated pathological processes differently affect functional cortical connectivity across cortical regions.

### S-maps, neurodevelopmental dynamics, and SZ

There are no systematic differences in synaptic density among the neocortical lobes in the adult human brain; however, synaptogenesis in the human neocortex appears to be regionally heterochronous [97]. These developmental differences persist longer for layers 2–3 that provide cortico-cortical connectivity. Considering that synaptogenesis occurs concurrently with dendritic and axonal growth/branching and with myelination, the state of connectivity across cortical areas must be significantly different in adolescence and in early adulthood when SZ symptoms emerge.

Recent neuroimaging studies provided a dynamic picture of heterochronous regional brain maturation. In general, cortical gray matter develops nonmonotonously: its volume increases during the first years of human life, but then, around puberty it starts to decrease. Judged from the gray-matter volume dynamics, the cortices likely containing the sources of the S-changes have clearly different developmental trajectories. Of them, the dorsal parietal cortex matures first; later, the frontal cortex follows. Parts of the temporal cortex that occupy the hemisphere convexity (e.g., superior and middle temporal gyri) continue to mature at least until young adulthood [98].

Furthermore, the dorsal aspects of the frontal and parietal cortices (compared to the lateral aspects of temporal and parietal cortices) seem to have different life-long dynamics. Neuroimaging across the life span showed significant decrease in the gray matter thickness in the dorsal cortex between 7 and 60 years of age, while in the temporal cortex it slowly increased until 30 years of age [99]. The same method applied to patients with early-onset SZ revealed an excessive loss of gray matter that started at the superior parietal cortex and spread to the temporal and prefrontal cortices [89].

Likely, both the gray-matter loss during development and its excessive decline in SZ are driven at least partially by the processes of synaptic and dendritic pruning. If this holds true, then the interplay between regionally heterochronous developmental processes and SZ-associated pathological processes might produce regionally distinct effects. Because of the life-long dynamics of the gray matter changes, this might be true not only for child-onset, but also for adult-onset SZ. In particular, the extended developmental trajectory of the frontal and temporal areas suggests their higher reserves of plasticity.

As noted by Innocenti and co-authors [100], reduced connectivity does not necessarily result in reduced functional coupling. Likely, partial loss of axonal and/or dendritic branches could provoke some abnormal reorganization of the remaining elements of neuropil. For instance, the residual axons might penetrate into vacated neuropil space, increase their number of boutons, and form anomalous contacts. Being implemented in the regions with a protracted developmental sequence like certain temporal and frontal areas, such a scenario would result in enhanced rather than reduced intra-areal coupling. However, similar initial pathological events could reduce coupling in the regions with a relatively short developmental trajectory like postcentral areas close to the interhemispheric margin.

Although such a hypothetical scenario adequately accounts for the regional specificity of synchronization changes shown here and fits the neurodevelopmental model of schizophrenia [100], [101], obviously it requires further investigation and independent confirmation with other methods. In particular, further inquiry into the nature of the regional specificity of connectivity changes in SZ is needed.
